# Desacetylmethylcolchicine in the Treatment of Myeloid Leukaemia

**DOI:** 10.1038/bjc.1956.75

**Published:** 1956-12

**Authors:** A. M. Jelliffe, J. E. MacIver


### DESACETYLMETHYLCOLCHICINE IN THE TREATMENT OF MYELOID LEUKAEMIA

A. M. JELLIFFE AND J. E. MACIVER

*From the Meyerstein Institute of Radiotherapy and the Bland-Sutton Institute of Pathology, The Middlesex Hospital, London, W.*1

Received for publication October 15, 1956

" COLCEMID " (desacetylmethylcolchicine) has been reported as a useful drug in the treatment of chronic myeloid leukaemia, without any serious side effects (Moeschlin, Meyer and Lichtman, 1953 ; Leonard and Wilkinson, 1955). Our experience has been limited to the treatment of four patients. The results in this small series appear to confirm that " colcemid " will control the disease successfully for a variable period of time in some, but not all, patients, but we do not consider that the toxic effects of this drug can be dismissed as unimportant.

### CASE HISTORIES

*Case* 1

A woman aged 58 was found to have chronic myeloid leukaemia in June 1953. Examination of the blood at that time showed a total white cell count of 500,000 per cu. mm., 87 per cent neutrophils and myeloid precursors. The disease responded to splenic irradiation. Further courses were given with gradually diminishing remissions until July 1955. She was then admitted to hospital as there had been a recent deterioration in her condition. The liver and spleen were enlarged down to the level of the umbilicus and in addition she had developed a generalised adenopathy. Blood examination showed a total white cell count of 57,000 per cu. mm., 67 per cent neutrophils, 12 per cent myeloid precursors ; haemoglobin 40 per cent. She failed to respond to blood transfusions, cortisone, 6-mercaptopurine and " colcemid " and died two months after admission.

*Case* 2

A man aged 60 was found to have polycythaemia in 1947. He received treatment with deep X-rays to the skeleton and later with P.32. The total white cell count was always high and by May 1955 it had risen to 62,000 per cu. mm., 92 per cent neutrophils, 5 per cent myeloid precursors. He was also having a considerable amount of pain from the very marked splenic enlargement. Bone marrow aspiration showed a generalised hyperplasia of all elements. He was given a course of deep X-rays to the spleen, which became impalpable, the white count falling to 40,000 per cu. mm. Six months later the spleen tip could be felt level with the umbilicus and he again complained of pain. The white cell count was 78,000 per cu. mm. but the haemoglobin remained at 108 per cent. The spleen was treated by irradiation three times in the next 18 months with a decreasing response on each occasion. The highest total white cell count recorded during this period was 126,000 per cu. mm. In November 1955 the patient strained his shoulder while gardening and was admitted two days later as an emergency with an extensive bruise over the right side of the chest. He was also complaining of severe pain from the enlarged spleen which was now palpable in the left iliac fossa. Investigation of the blood showed a haemoglobin of 60 per cent; total white blood cells 146,000 per cu. mm., 83 per cent neutrophils, 10 per cent neutrophil precursors; platelet count 525,000 per cu. mm. He was treated with "colcemid" with an immediate fall in the white cell count and a rise in the haemoglobin, with some diminution in the size of the spleen. The white count rose rapidly when the daily dose of "colcemid" was reduced from

FIG. 1.—Case 2: Effect of "colcemid" on total white cells. Rapid response on reducing daily dose from 5 mg. to 3 mg.

5 mg. to 3 mg. (Fig. 1). The patient died of hypostatic pneumonia before any further observations could be made.

*Case* 3

A 30-year-old woman was investigated elsewhere in February 1952 for abdominal discomfort of six months' duration. She was found to have chronic myeloid leukaemia with a total white blood cell count of 223,000 per cu. mm. and a haemoglobin of 68 per cent. A satisfactory remission followed a course of deep X-ray therapy to the spleen. Thirteen months later the white cell count had risen to 131,000 per cu. mm. and it again fell rapidly with irradiation. Between

January 1954 and March 1955, three courses of deep X-ray therapy were necessary to control her symptoms and blood count. Each remission was of progressively shorter duration, and it was decided to try the effect of myleran. For 30 days 4 mg. daily were given with some effect on the blood count. The patient was then found to be pregnant. It was thought that this might lead to a deterioration of her leukaemia and, as she already had two children, a hysterotomy was combined with sterilisation. Three months later the total white cell count had risen to 140,000 per cu. mm. and she was again having pain in the left subcostal region. Deep X-ray therapy to the spleen produced a rapid response but the remission was short, and three months later the total white cell count had

FIG. 2.—Case 3: Resistance to "colcemid" in case responding to X-ray therapy and myleran.

increased to 170,000 per cu. mm. She felt very ill, had lost weight and was again complaining of pain in the splenic region. Treatment with "colcemid" was started with 2 mg. twice daily by mouth. The dose was gradually increased, without any improvement, until a daily total of 10 mg. was reached. At this level, the white cell count showed a slight response, but unfortunately this was not maintaned. The daily dose was then gradually raised to 13 mg. without any benefit. The total white cell count showed no real decrease, the haemoglobin level did not rise appreciably and the spleen remained unaltered in size. The patient continued to feel lethargic and generally unwell, and she became extremely depressed, worrying a great deal over her failure to respond to treatment. It was decided to give myleran a further trial, using a larger initial dose than 4 mg. daily, as this had not proved very effective previously. A satisfactory remission occurred and the total white cell count fell rapidly with a simultaneous rise in the haemoglobin (Fig. 2).

*Case* 4

A man aged 37 was admitted to hospital as an emergency in September 1955 with severe pain in the left upper abdomen and shoulder, which had been present for three days. He was found to have a firm tender spleen, enlarged to just above the umbilicus. Examination of the blood showed a haemoglobin of 67 per cent and a total white cell count of 148,000 per cu. mm. with a differential count typical of chronic myeloid leukaemia, and the diagnosis was confirmed by bone marrow aspiration. Deep X-ray therapy was given to the spleen, which became impalpable, and the blood count responded satisfactorily. Five months later the total white blood count had increased to 142,000 per cu. mm. and the haemoglobin had fallen. At his next hospital attendance four weeks later he complained once more of left-sided pain and extreme lethargy. It was decided to try the effect of " colcemid ". A satisfactory initial response occurred, and this was maintained on 6 mg. daily in divided doses. The spleen, which had again enlarged to the level of the umbilicus, became painless, but there was no decrease in size during the treatment. His appetite and weight improved and he felt increasingly fit. Unfortunately, the platelet count fell slowly to 90,000 per cu. mm., and the drug was stopped after a symptom-free remission of five months.

Two weeks later, the total white count had risen to 110,000 and the haemoglobin was falling. The patient felt unwell and the original pain returned. Further irradiation was given to the spleen with an immediate fall in the total white cell count and a simultaneous subjective improvement. The spleen shrank a little but the response was disappointing. This remission lasted only two months. The platelet count had by now risen to over 200,000 per cu. mm. and it was decided to try " colcemid " once more. The total white cell count fell rapidly but there was no increase in the haemoglobin level. The drug was stopped for 12 days over Christmas and was recommenced on December 29. Two days later the patient felt extremely ill and his hair suddenly began to fall out. Three days after this he attended for follow-up examination. " Colcemid "was stopped and he was admitted to hospital. There was by this time a severe generalised loss of scalp hair and quantities of hair could easily be removed by gentle combing. He had also noticed thinning of the hair over the rest of the body. His general condition was poor, with pitting oedema of both ankles. The spleen was palpable below the level of the umbilicus and the liver was enlarged to just above this level.

The following relevant investigations were carried out. Blood count : Hb 30 per cent. W.B.C. 107,000 per cu. mm. Neutrophils 17 per cent. Lymphocytes 6 per cent. Monocytes 2 per cent. Eosinophils 1 per cent. Basophils 2 per cent. Metamyelocytes 2 per cent. Myelocytes 43 per cent. Premyelocytes 2 per cent. Myeloblasts 25 per cent. 38 nucleated red cells/100 white cells. Platelets 77,000 per cu. mm. Bone marrow aspiration : differential count identical with peripheral blood. Bone marrow biopsy : Acellular marrow with some increase of fibrous tissue. Serum bilirubin : 1·1 mg. per cent. Reticulocytes : 1 per cent. Coombes test : negative.

A diagnosis of generalised marrow aplasia was made, and further treatment with any cytotoxic drug was thought to be contra-indicated. Repeated blood transfusions, prednisone and cortisone were given to try to postpone the inevitable outcome. Small doses of X-rays were given to the spleen in an attempt to reduce its size and relieve the patient's discomfort but none of these measures appeared to affect his rapid downhill progress. Terminally the total white cell count rose to over 400,000 per cu. mm., 67 per cent of which were myeloid precursors, and he finally died six weeks after admission to hospital (Fig. 3).

Four days before his death he complained of variable left-sided lower abdominal pain, radiating down the left leg above the knee, and an area of anaesthesia to pin prick and light touch was found over the anterior surface of the affected thigh. The spleen had by then enlarged until it completely filled the

Fig. 3.—Case 4 : Control by " colcemid ", followed by rapid relapse.

left iliac fossa, and these findings were thought to be due to nerve pressure or actual invasion by the leukaemic process. The autopsy proved these assumptions to be incorrect. During the first two weeks of his stay in hospital, depilation continued at a gradually diminishing rate and over the last week of his life it was thought that there had been some slight regrowth of hair.

Post-mortem examination showed that the spleen was grossly enlarged, weighing 7 lb., and its surface was mottled by old and recent infarcts. The liver weighed 8 lb. and was pale, the macroscopical appearance suggesting myeloid leukaemia. The bone marrow in all the bones examined was pale. On the left lateral and postero-lateral wall of the abdominal cavity, there was a large smooth swelling, pushing the spleen down and the kidney upwards. When the swelling was incised about 2½ pints of semi-solid blood clot was evacuated.

On microscopical examination, the typical changes of myeloid leukaemia were found in the liver, spleen and kidneys. The marrow from all areas was highly cellular. Megakaryocytes and red blood cells were completely absent, and the cells consisted almost entirely of primitive white cells. The testicles showed a marked maturation defect and skin from both the scalp and the scrotum showed a number of normal hair follicles.

### DISCUSSION

Case 1 did not respond to " colcemid " or to any form of treatment. This is not surprising, in view of the advanced stage of the disease.

Case 2 showed a marked fall in the white cell count, associated with a rapidly rising haemoglobin, and some decrease in the size of the spleen. Reduction of the dose of " colcemid " led to an immediate rise in the white cell count. The unexpected death of the patient from bronchopneumonia unfortunately prevented any further observations. This complication cannot be attributed to the drug.

Case 3 proved resistant to " colcemid " in doses of up to a maximum of 13 mg. daily. Leonard and Wilkinson (1955) refer to two out of a total of eight patients with chronic myeloid leukaemia who were resistant to " colcemid " up to a maximum dose of 10 mg. daily. Both their cases were also resistant to myleran and 6-mercaptopurine. No mention is made if they responded to radiotherapy, either before or after " colcemid " therapy. Our case was resistant to " colcemid " but responded to splenic irradiation and to myleran. Resistance to " colcemid " does not therefore mean that other methods of treatment will be unsuccessful.

Case 4 developed loss of scalp hair, which was referred to by Leonard and Wilkinson (1955) as a complication in one of their cases. Their patient also noticed some dysphagia and scrotal tenderness, and the hair loss occurred at the height of the depression of the white cells. No mention is made of regrowth of the hair, and treatment with " colcemid " was abandoned. Our patient developed severe generalised alopecia of the whole scalp and loss of hair in the axillae and on the eyebrows and scrotum. It occurred during the second course of " colcemid " and we are unable to relate this directly to the degree of depression of the white cell count. Although the count at this time was low, the fall was not as marked as it had been on previous occasions during the first course of treatment. No dysphagia or scrotal irritation was noticed and there was no eosinophilia.

This complication has been noted previously during the treatment of human cancer with large doses of colchicine (Seed, Slaughter and Limarzi, 1940). A woman aged 55 with advanced carcinoma of the breast was given intramuscular injections of the drug with some temporary improvement in the tumour. On the 21st day of the treatment her scalp hair suddenly started to fall out in handfuls and in four days she was totally bald. She continued to have injections of colchicine for a further six weeks and, in spite of this, complete regrowth of the scalp hair occurred in five months. If the alopecia produced by " colcemid " is similar, regrowth of the hair may be expected and this was suggested by the findings in our case. It is possible that regrowth of the hair might occur during the continued administration of " colcemid ", but it would require a certain amount of courage to test out this hypothesis.

Before the death of the patient it was thought that marrow failure had developed as a result of treatment, and for this reason no further chemotherapy was given. Although no case of marrow aplasia following the use of " colcemid " had been reported previously, this complication was attributed to the drug rather than to the natural progress of the disease. The alternative diagnosis of myelosclerosis was considered unlikely as the original bone marrow examination had shown the typical findings of chronic myeloid leukaemia. Some authors believe that myeloid leukaemia and myelosclerosis are one and the same disease (Heller, Lewisohn and Palin, 1947). In fact very few untreated cases have been recorded where the original bone marrow examination showed frank myeloid leukaemia—to be followed at a later date by the development of myelosclerosis.

Post-mortem examination showed that failure of the marrow was due to extensive invasion by primitive forms of the myeloid series. All marrow specimens examined showed these changes, emphasising the danger of relying on the examination of one sample of bone marrow. In light of this knowledge, the response of the leukaemia to the treatment was reviewed and it appeared that " colcemid " was in fact controlling the disease. When the drug was finally stopped, the total count rapidly rose and large numbers of primitive cells of the myeloid series appeared in the peripheral circulation. An alternative explanation that might be offered for the final exacerbation is that the " colcemid " stimulated the leukaemia into an acute phase. Similar charges have been made against deep X-ray therapy, and irradiation is still sometimes blamed when chronic leukaemia enters a terminal acute phase or when the changes of lymphatic leukaemia become manifest in the course of progress of a case of lymphosarcoma. There is very little knowledge of the natural history of untreated leukaemia and without control series of cases, such criticisms must be without foundation.

The chemotherapeutic control of chronic myeloid leukaemia has been achieved for long periods of time without serious complications (Galton and Till, 1955). When chemotherapeutic agents are used it is important that they should be as free from unpleasant side effects, as effective and as easily administered as X-ray therapy. From the small amount of evidence available, we do not consider that " colcemid " is as free from distressing side effects or more effective than X-ray therapy or myleran. We do not think it should be used in the treatment of chronic myeloid leukaemia unless the patient has become refractory both to irradiation and to myleran. Leonard and Wilkinson (1955) refer to the dangers of myleran but it would appear that these have been almost entirely eliminated by the adoption of more suitable dosage schedules (Galton and Till, 1955). " Colcemid " seems to be contra-indicated particularly in the treatment of women, but it might be used with less anxiety if the patient is a man who is already bald. The drug is effective in controlling some cases of chronic myeloid leukaemia and so far there is no evidence that serious marrow depression follows its use.

### SUMMARY

An account is given of the treatment of myeloid leukaemia with desacetylmethylcolchicine. The drug appears to be an additional effective weapon in the control of some patients with this disease.

There has been as yet no recorded case of dangerous bone marrow depression during " colcemid " administration, and the only serious toxic effect encountered has been depilation, which is probably reversible.

Depilation is a distressing complication and we do not think " colcemid " should be used in the treatment of chronic myeloid leukaemia, unless the patient no longer responds to splenic irradiation and to myleran.

We wish to thank Professor B. W. Windeyer and Miss M. D. Snelling for allowing us to report patients under their care. Part of the expenses of this investigation has been defrayed by the British Empire Cancer Campaign.

### REFERENCES

GALTON, D. A. G. AND TILL, M.—(1955) *Lancet*, i, 425.
HELLER, E. L., LEWISOHN, M. G. AND PALIN, W. E.—(1947) *Amer. J. Path.*, **23**, 327.
LEONARD, B. J. AND WILKINSON, J. F.—(1955) *Brit. med. J.*, i, 874.
MOESCHLIN, S., MEYER, H. AND LICHTMAN, A.—(1953) *Schweiz. med. Wschr.*, **83**, 990.
SEED, L., SLAUGHTER, D. P. AND LIMARZI, L. R.—(1940) *Surgery*, **7**, 696.


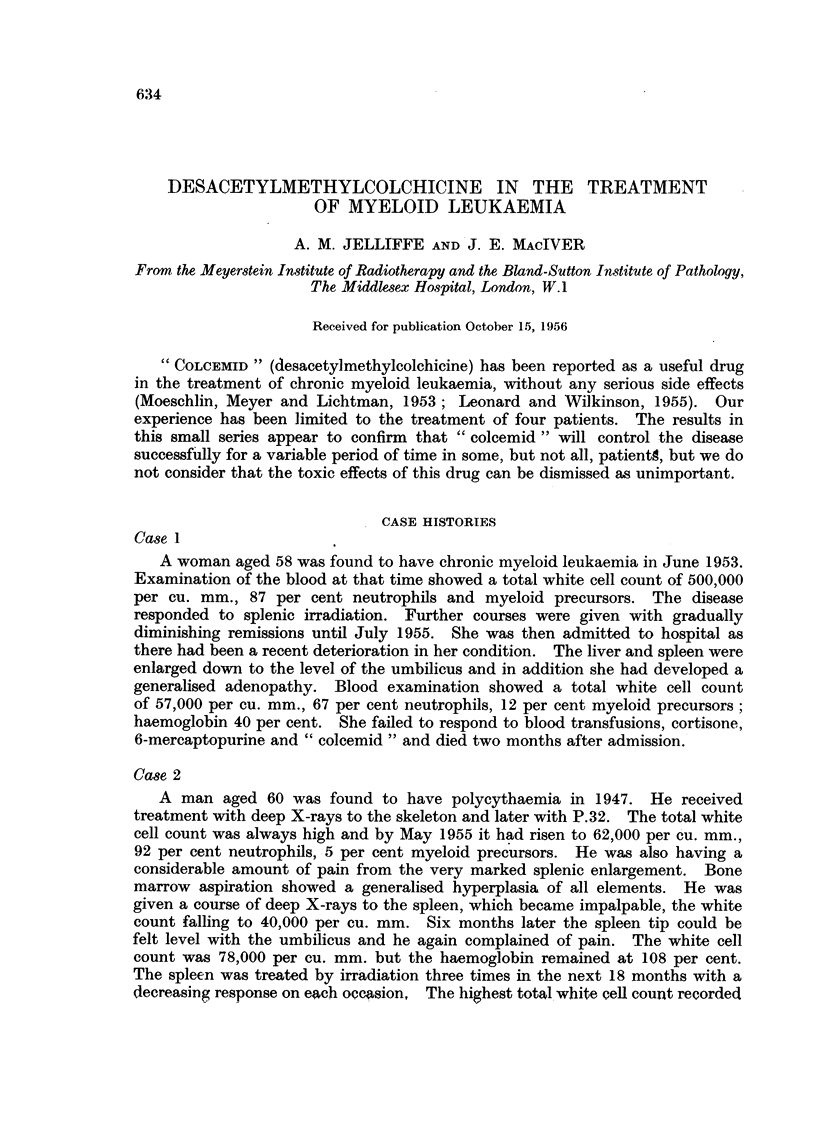

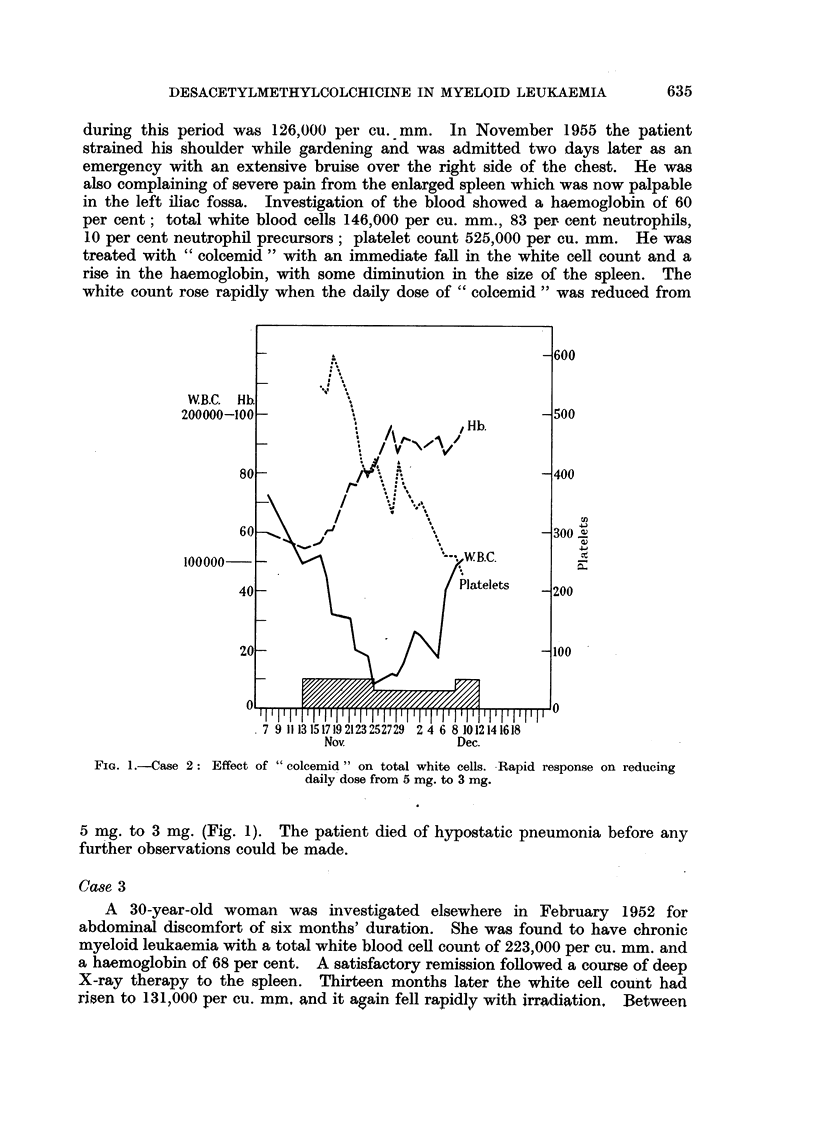

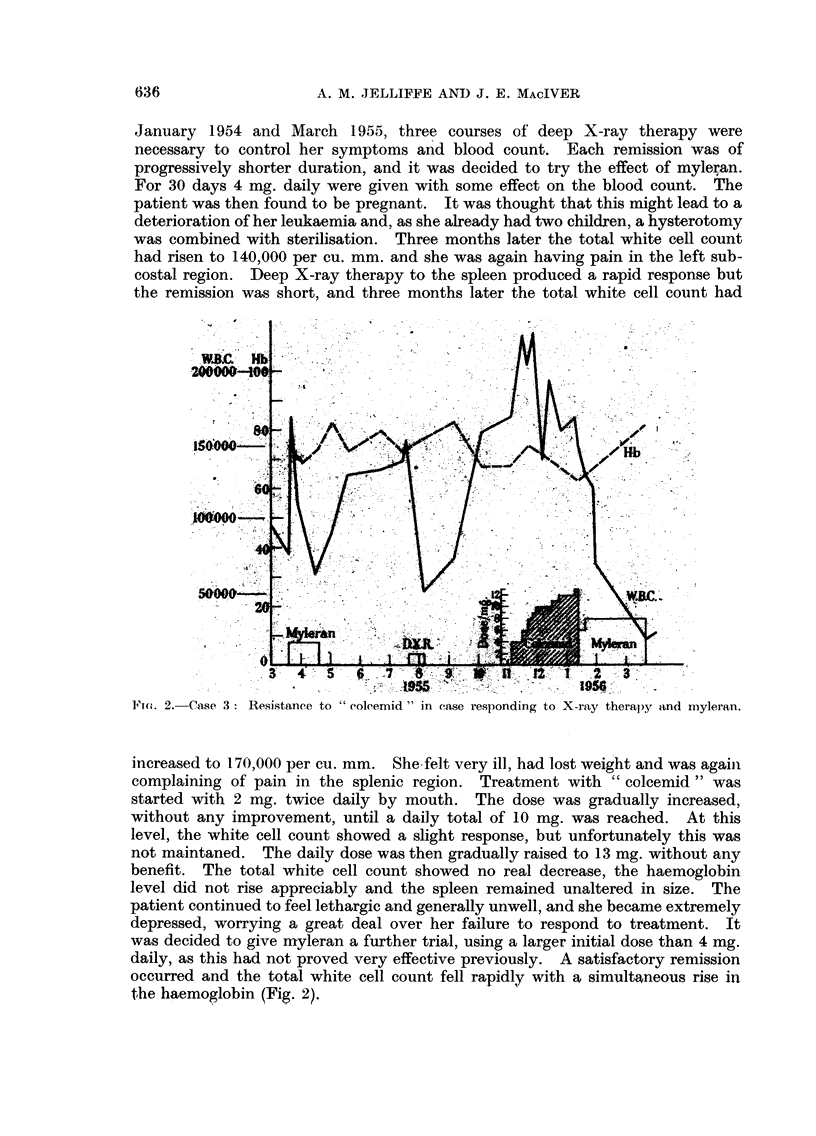

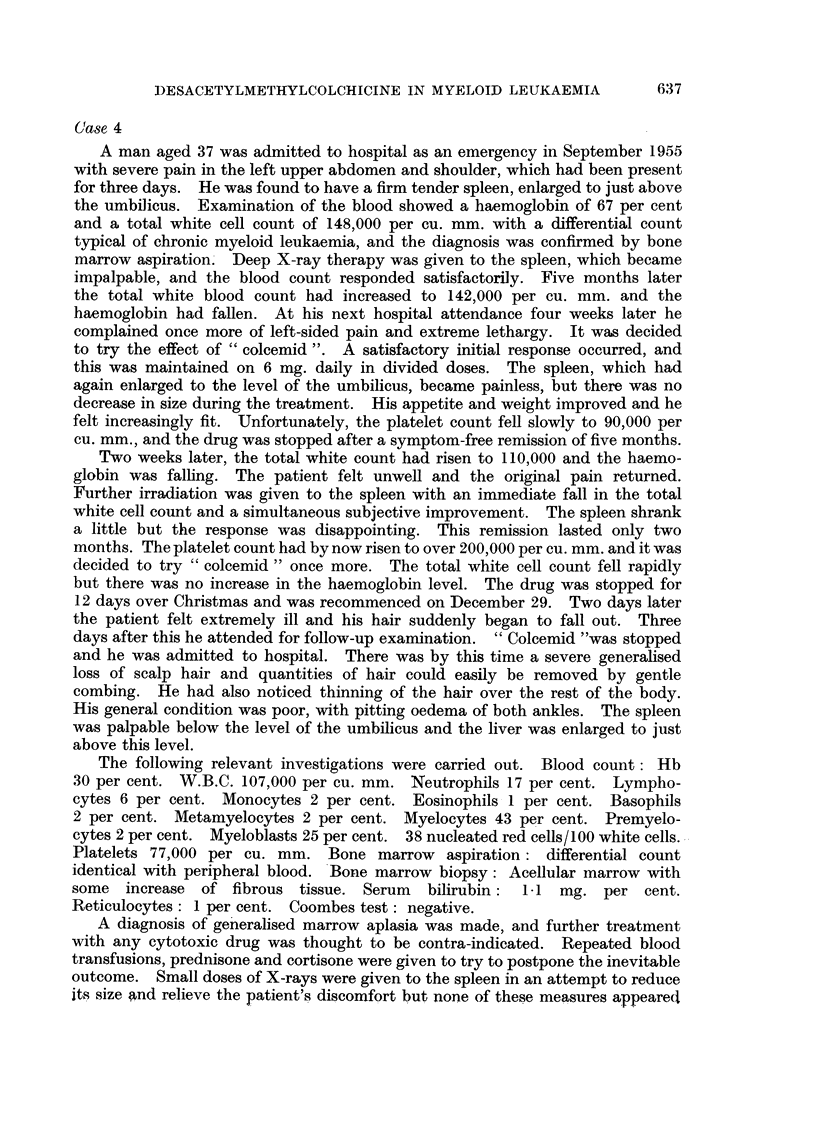

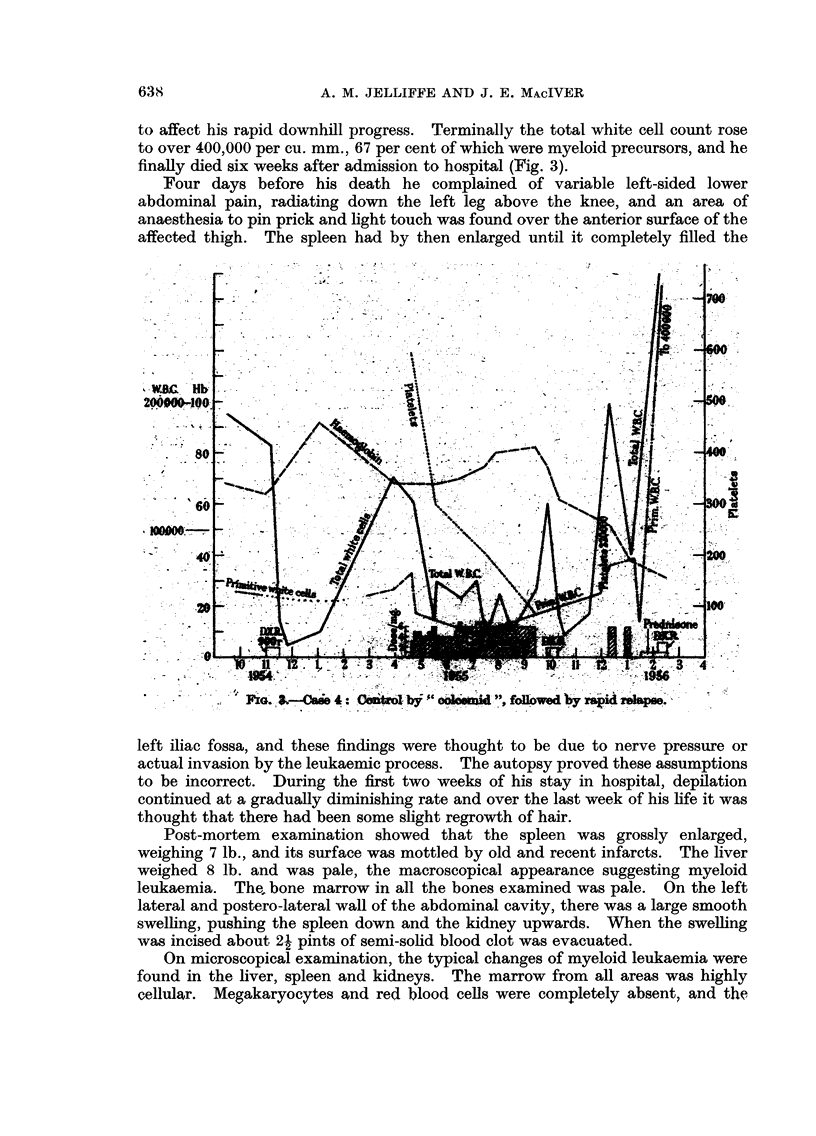

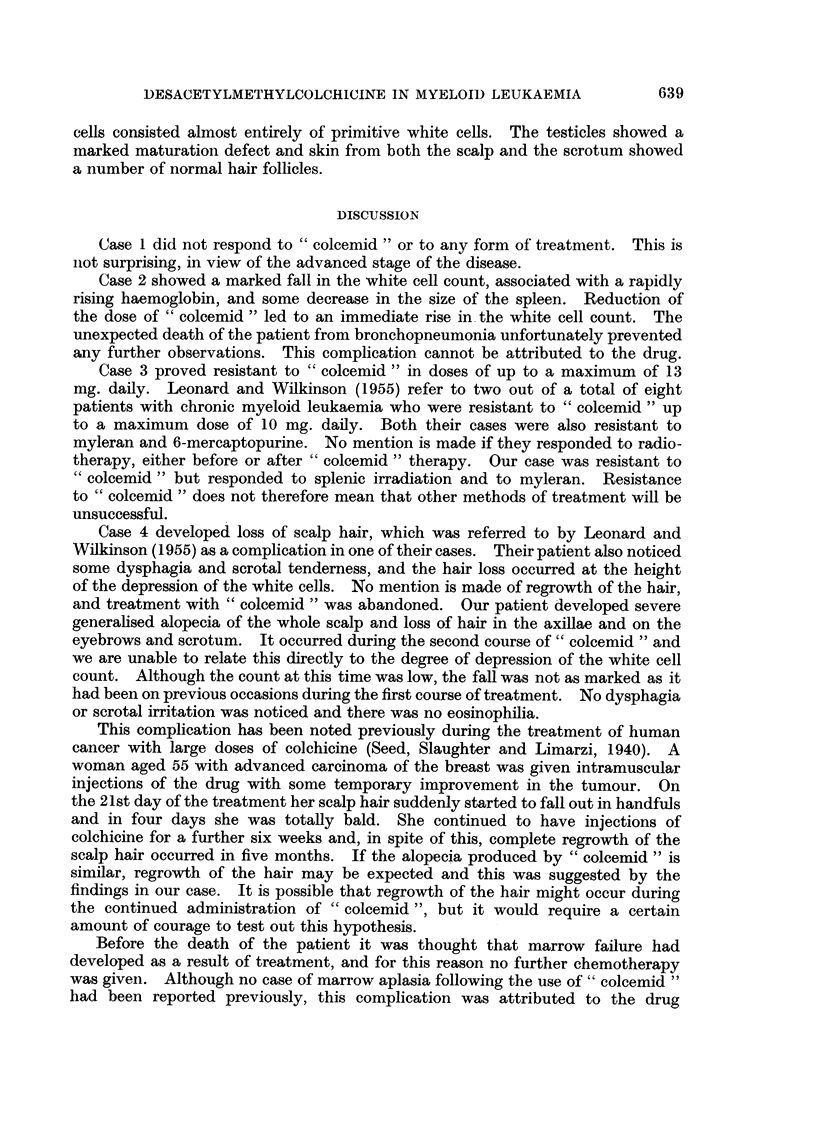

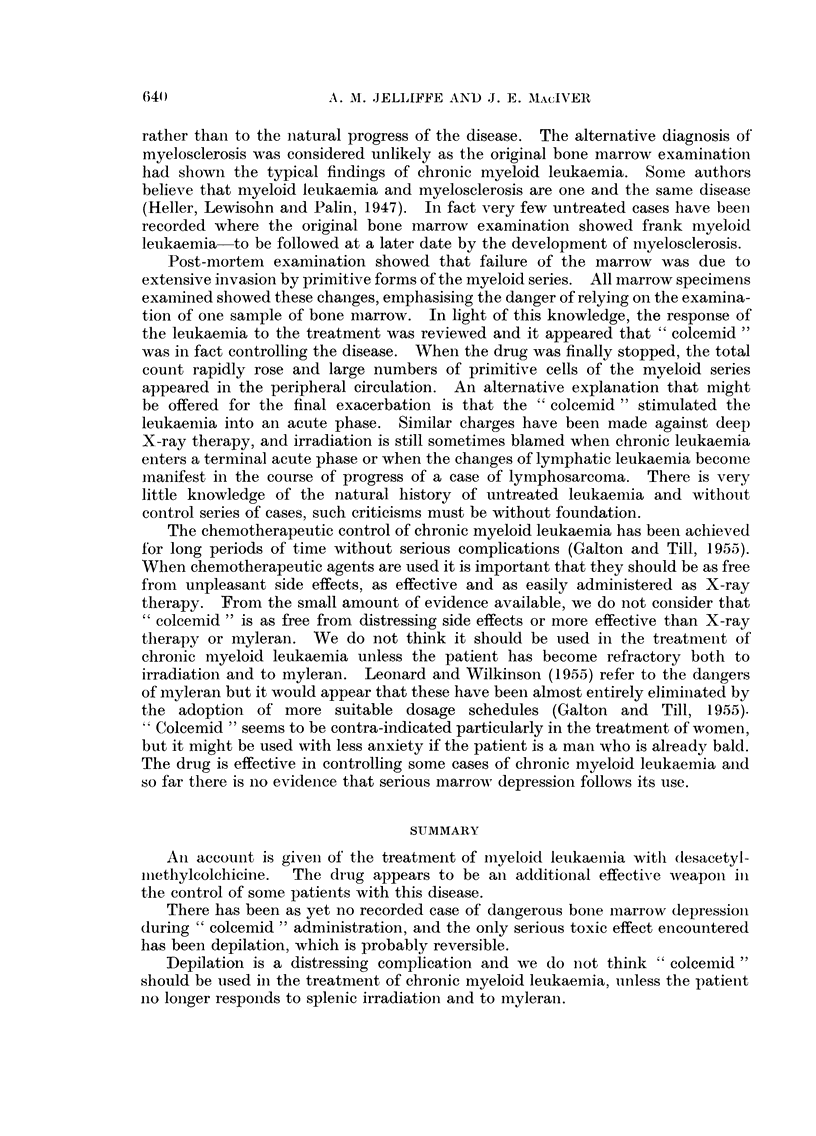

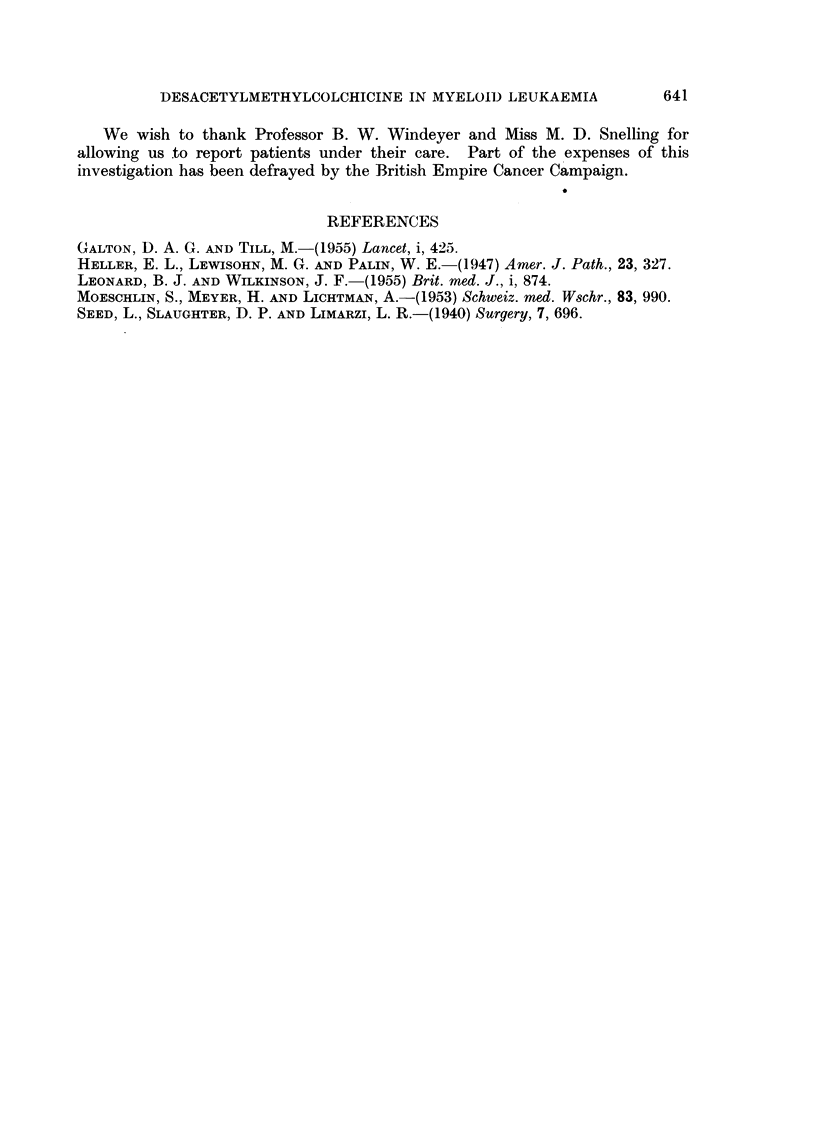


## References

[OCR_00347] GALTON D. A., TILL M. (1955). Myleran in chronic myeloid leukaemia.. Lancet.

[OCR_00349] Heller E. L., Lewisohn M. G., Palin W. E. (1947). Aleukemic Myelosis: Chronic Nonleukemic Myelosis, Agnogenic Myeloid Metaplasia, Osteosclerosis, Leuko-Erythroblastic Anemia, and Synonymous Designations.. Am J Pathol.

[OCR_00350] LEONARD B. J., WILKINSON J. F. (1955). Desacetylmethylcolchicine in treatment of myeloid leukaemia.. Br Med J.

[OCR_00352] MOESCHLIN S., MEYER H., LICHTMAN A. (1953). Ein neues Colchicum-Nebenalkaloid (Demecolcin Ciba) als Cytostaticum myeloischer Leukämien.. Schweiz Med Wochenschr.

